# Delayed Tracheal Perforation Following Thyroid Lobectomy: A Postoperative Diagnostic Challenge

**DOI:** 10.1016/j.aed.2025.06.005

**Published:** 2025-07-01

**Authors:** Katrina Ann H. Corpuz, Jeyavishnupriya Gopalakrishnan, Heng Yeh, Juan J. Delgado Hurtado

**Affiliations:** 1 Bassett Medical Center, Department of Medicine, Cooperstown, New York; 2 Bassett Medical Center, Division of Endocrinology, Cooperstown, New York

**Keywords:** postoperative complication, thermal injury, thyroidectomy, thyroid lobectomy, tracheal perforation

## Visual Vignette

### Case Presentation

A 50-year-old female with a history of dyslipidemia, esophageal reflux presented with choking sensation and shortness of breath. She also reported to have worsening neck swelling with pressure sensation, especially while lying flat. She underwent right thyroid lobectomy for a symptomatic 5.6 × 4.0 × 2.0 cm mixed cystic and isoechoic thyroid nodule 11 days prior to arrival. The histopathology revealed a benign follicular adenoma. Postoperative recovery was initially unremarkable, aside from a small hematoma that resolved conservatively. Examination revealed prominent right-sided neck swelling with firm, nonpulsatile mass under the healing horizontal neck incision with palpable subcutaneous crepitus. There was no overlying erythema, drainage, or dehiscence. Patient remained hemodynamically stable with normal oxygen saturation. Computed tomography (CT) of the neck revealed a large air collection in the right thyroidectomy bed measuring 9.5 × 7.0 × 6.0 cm (Fig).

**Fig:**
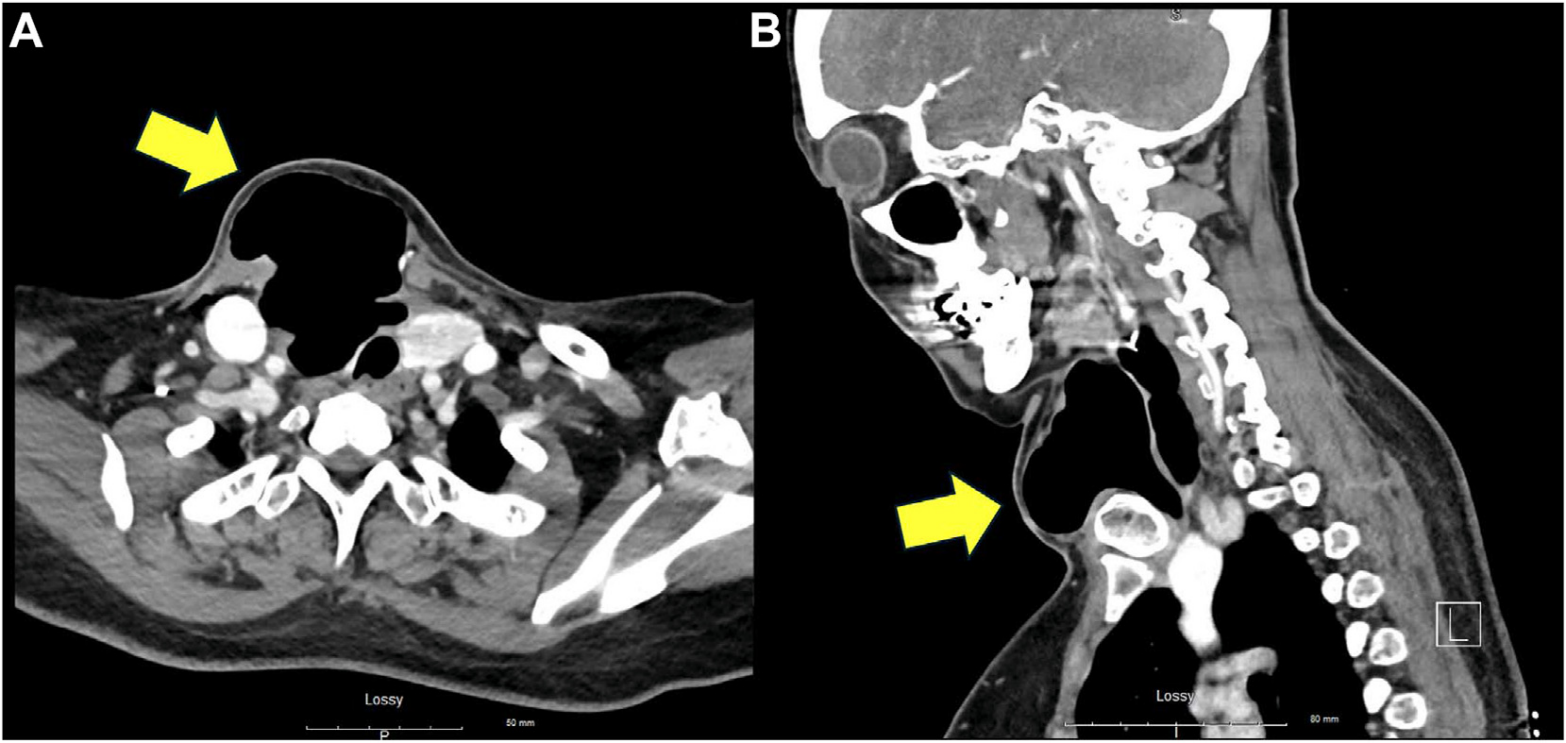
Axial (*A*) and sagittal (*B*) CT neck images showing a 9.5 × 7.0 × 6.0 cm air collection (yellow arrows) in right thyroidectomy bed. *CT* = computed tomography.

## What is the diagnosis?

### Diagnosis

Delayed tracheal perforation secondary to thermal injury during thyroid lobectomy. Tracheal injuries following thyroidectomy, although rare, can lead to significant morbidity if not promptly identified. Thermal injuries from electrocautery represent an uncommon but recognized mechanism causing delayed tracheal perforation, often presenting days to weeks postoperatively.^1^ Thermal injury to the trachea during thyroidectomy can lead to devascularization of the tracheal wall, resulting in necrosis and subsequent perforation. This process may be exacerbated by postoperative factors such as infection or increased intrathoracic pressure from coughing or sneezing.^2^ Clinical features typically include progressive neck swelling, subcutaneous emphysema, dysphagia, and respiratory symptoms exacerbated by coughing or positional changes.^1^ Subcutaneous emphysema palpated on physical examination strongly suggests underlying tracheal injury, warranting immediate imaging and surgical consultation.^3^ CT imaging is the preferred modality for evaluating subcutaneous emphysema and identifying the extent of tracheal injury. Prompt recognition and management are crucial to prevent severe complications, including mediastinitis and airway compromise.^2^

## Statement of Patient Consent

Informed consent was obtained from the patient for the publication of this case and associated images.

## Disclosure

The authors have no conflicts of interest to disclose.
